# Whole-Genome Sequencing and Analysis of the White-Rot Fungus *Ceriporia lacerata* Reveals Its Phylogenetic Status and the Genetic Basis of Lignocellulose Degradation and Terpenoid Synthesis

**DOI:** 10.3389/fmicb.2022.880946

**Published:** 2022-05-24

**Authors:** Zhitao Mao, Ping Yang, Huanhuan Liu, Yufeng Mao, Yu Lei, Dongwei Hou, Hongwu Ma, Xiaoping Liao, Wenxia Jiang

**Affiliations:** ^1^Biodesign Center, Key Laboratory of Systems Microbial Biotechnology, Tianjin Institute of Industrial Biotechnology, Chinese Academy of Sciences, Tianjin, China; ^2^Tianjin Key Laboratory for Industrial Biological Systems and Bioprocessing Engineering, Tianjin Institute of Industrial Biotechnology, Chinese Academy of Sciences, Tianjin, China; ^3^National Technology Innovation Center of Synthetic Biology, Tianjin, China; ^4^Tianjin Institute of Industrial Biotechnology, Chinese Academy of Sciences, Tianjin, China; ^5^State Key Laboratory of Food Nutrition and Safety, Tianjin University of Science and Technology, Tianjin, China

**Keywords:** *Ceriporia lacerata*, *de novo* genome sequencing, phylogenetic analysis, lignin degradation, terpenoid biosynthesis, comparative genomics

## Abstract

*Ceriporia lacerata* is an endophytic white-rot fungus that has lignocellulolytic and terpenoid-biosynthetic abilities. However, little is known about the genomic architecture of this fungus, even at the genus level. In this study, we present the first *de novo* genome assembly of *C. lacerata* (CGMCC No. 10485), based on PacBio long-read and Illumina short-read sequencing. The size of the *C. lacerata* genome is approximately 36 Mb (N50, 3.4 Mb). It encodes a total of 13,243 genes, with further functional analysis revealing that these genes are primarily involved in primary metabolism and host interactions in this strain’s saprophytic lifestyle. Phylogenetic analysis based on ITS demonstrated a primary evolutionary position for *C. lacerata*, while the phylogenetic analysis based on orthogroup inference and average nucleotide identity revealed high-resolution phylogenetic details in which *Ceriporia, Phlebia*, *Phlebiopsis*, and *Phanerochaete* belong to the same evolutionary clade within the order Polyporales. Annotation of carbohydrate-active enzymes across the genome yielded a total of 806 genes encoding enzymes that decompose lignocellulose, particularly ligninolytic enzymes, lytic polysaccharides monooxygenases, and enzymes involved in the biodegradation of aromatic components. These findings illustrate the strain’s adaptation to woody habitats, which requires the degradation of lignin and various polycyclic aromatic hydrocarbons. The terpenoid-production potential of *C. lacerata* was evaluated by comparing the genes of terpenoid biosynthetic pathways across nine Polyporales species. The shared genes highlight the major part of terpenoid synthesis pathways, especially the mevalonic acid pathway, as well as the main pathways of sesquiterpenoid, monoterpenoid, diterpenoid, and triterpenoid synthesis, while the strain-specific genes illustrate the distinct genetic factors determining the synthesis of structurally diverse terpenoids. This is the first genomic analysis of a species from this genus that we are aware of, and it will help advance functional genome research and resource development of this important fungus for applications in renewable energy, pharmaceuticals, and agriculture.

## Introduction

*Ceriporia lacerata* was identified as a new species that causes white rot in both angiosperm and gymnosperm wood in 2003 (Suhara et al., 2003). In recent years, *C. lacerata* has drawn much attention as an important microbial resource with great potential in the agricultural, pharmaceutical, and renewable energy industries, due to its versatile arsenal of enzymes for the pretreatment of lignocellulosic biomass (Lee et al., 2007), biodegradation of polycyclic aromatic compounds (PACs) (Al-Hawash et al., 2020), decolorization of synthetic dyes (Xu et al., 2008; Lin et al., 2011; Wang et al., 2017), and biosynthesis of terpenoids (Shan et al., 2012; Ying et al., 2013a,b, 2014a,b; Zhao et al., 2013), as well as being able to improve the soil and crop performance by phosphorus mobilization (Yin et al., 2021).

Generally, *Ceriporia* spp. are saprotrophs and engage in a complicated web of interactions with their host plants, such as *Huperzia serrata* (Cui et al., 2006; Dai, 2012). *Ceriporia* produces ligninolytic enzymes that can destroy the lignin components of the plant cell wall. The secretory lignin-modifying enzymes, including laccases, lignin peroxidases, and manganese-dependent peroxidases, allow *Ceriporia* to get access to plant polysaccharides as carbon sources (Mkel et al., 2021). Due to the structural similarity between lignin and PACs, *Ceriporia* was also successfully applied to degrade polychlorinated biphenyls (PCBs) present in soil and sediments (Mori and Kondo, 2002; Suhara et al., 2003; Seifried and Temelli, 2009; Hong et al., 2012, 2013) and various industrial dyes in wastewater (Choi et al., 2013; Cerrón et al., 2015; Lee et al., 2015). In addition to its ligninolytic ability, Tang et al. (2010) evaluated the carbohydrate-degradation ability of *C. lacerata* F1 by measuring the changes of chemical composition, structural modifications, and their susceptibility to enzymatic saccharification in degraded wood, revealing that *C. lacerata* has endoglucanase (EG) and filter paper cellulase activities. Notably, *Ceriporia* can also synthesize a series of secondary metabolites and has gained attention as a microbial source of biocatalysts for the biotransformation of natural terpenoid products (Shan et al., 2012; Liu et al., 2013; Ying et al., 2013b, 2014b; Zhao et al., 2013), such as ceriponols A∼K (Ying et al., 2013b), ceriponols L∼M (Shan et al., 2012), and ceriponol P (Ying et al., 2014b), among which ceriponols F, G and K exhibited cytotoxicity against all tested human cancer cell lines, indicating a pharmaceutical potential in this species. However, further research on this fungus necessitates detailed genetic knowledge, which is dependent on a well-characterized, foundational reference genome. Unfortunately, little information concerning the genome of *Ceriporia* has been reported to date.

Taxonomically, *Ceriporia* is traditionally classed into the family Irpicaceae (order Polyporales) with other 14 genera based on morphological, physiological, and biochemical characteristics (Jia et al., 2013). This genus produces resupinate basidiocarps with a variety of pore-like surface colors, a monomitic hyphal structure with simple septa on generative hyphae, and thin-walled hyaline, and usually cylindrical to oblong-ellipsoid basidiospores (Jia et al., 2013). However, these phenotypic features have limitations in the identification of fungal species due to their susceptibility to convergent evolution, reduction, or disappearance (Zhang et al., 2017). Recently, the taxonomy and phylogeny of the genus *Ceriporia* has been revised based on molecular phylogeny of mitochondrial small subunit rDNA (mt SSU) (Kim and Jung, 1999; Lomsadze et al., 2014), the nuclear rDNA internal transcribed spacer ITS1-5.8S-ITS2 (ITS) region, nuclear 28S rDNA (28S), and the gene encoding the largest subunit of RNA polymerase II (*rpb1*) (Jia et al., 2013; Floudas and Hibbett, 2015; Miettinen et al., 2016; Justo et al., 2017; Yuan et al., 2017), but due to insufficient phylogenetic information and gene-specific noise, single or a few loci (multilocus) frequently yield incongruent phylogenies, resulting in several weakly supported nodes. Hence, a denser sampling of larger and identical gene sets across the genome is required to advance *Ceriporia* phylogeny (Binder et al., 2013; Zhang et al., 2017).

In our previous work, a *C. lacerata* strain isolated from the Changbai Mountain in Northeast China and deposited as CGMCC No. 10485, was able to grow rapidly on non-sterilized lignocellulosic substrates (Shao et al., 2016). In order to provide genomic information to further understand and develop this fungal resource, *C. lacerata* CGMCC No. 10485 was subjected to *de novo* genome sequencing and annotation in this study. Based on this, we addressed its molecular phylogeny and the genetic basis of lignocellulose degradation and terpenoid synthesis. To our best knowledge, this is the first fully annotated genome sequence for this genus, which will promote the mining of functional genetic elements of this strain and its future development as a genetic resource.

## Materials and Methods

### Strain, Culture Conditions, and Sample Collection

The mycelium of *C. lacerata* CGMCC No. 10485 was stored at 4°C after initial cultivation on PDA solid medium for 7 days at 25°C. A small amount of the mycelium from slant culture was transferred into a 500-mL conical shake flask containing 150 mL first-order seed culture medium (soluble starch 20 g/L, spray-dried corn steep liquor 6 g/L, KH_2_PO_4_ 1 g/L, sterilized at 121°C for 20 min), then cultivated for 3.5 days in a rotary shaker at 150 rpm and 25°C.

Then, 7.5 mL of the first-order seed culture was used to inoculate a 500-mL flask containing 150 mL of second-order seed culture medium (glucose 80 g/L, spray-dried corn steep liquor 8 g/L, KH_2_PO_4_ 5 g/L, sterilized at 115°C for 30 min) and grown in a rotary shaker at 150 rpm and 25°C for sequential sampling on the 2nd day (lag phase, denoted as CL1), day 2 (early logarithmic phase, CL2), day 5 day (logarithmic phase, CL3), and day 8 (stationary phase, CL4) (Growth characteristics were described in Supplementary Figure 1). The mycelium pellets from different culture stages were separated from the medium by filtration, shock-frozen in liquid nitrogen, and stored at −80°C for RNA/DNA isolation and sequencing.

### DNA Isolation and Sequencing

The genomic DNA was isolated using the DNeasy Plant Mini Kit (QIAGEN) according to the manufacturer’s instruction. PacBio sequencing was performed by Novogene (Tianjin, China) on a PacBio RS platform with one SMART cell, which generated long-read data. In addition, another run of Illumina sequencing was performed on a NovaSeq 6000 platform (standard 2 × 150 paired-end libraries), which generated short-read data. All the raw data in FASTQ format were processed to get clean reads by removing adapters, N bases, and low-quality bases.

### RNA Isolation and Sequencing

Total RNA collected at CL1-CL2-CL3-CL4 was extracted using the RNAprep Pure Cell/Bacteria Kit (TIANGEN, China). The integrity of the total RNA was assessed using 1.0% agarose gel electrophoresis. The concentration and purity were determined using a NanoDrop 2000 spectrophotometer. Total RNA (1 μg) was treated with the QIAseq*™* FastSelect−5S/16S/23S Kit (QIAGEN) to remove rRNA, and cDNA libraries were prepared using the QIAseq*™* Stranded Total RNA Lib Kit according to the manufacturer’s instructions. Illumina sequencing was performed on a NovaSeq 6000 platform by Novogene (Tianjin, China). Raw data were filtered to obtain the high-quality RNA-seq data using the same procedure described above for genomic short-read data. The raw sequencing data (both genomic and transcriptomic) have been submitted to the NCBI Sequence Read Archive under BioProject PRJNA804482 (link).^1^

### Genome Assembly and Functional Annotation

Long-read data generated by the PacBio platform were *de novo* assembled using CANU 2.1 as described previously (Koren et al., 2017). Briefly, the long reads were corrected, trimmed, and then assembled into contigs. Subsequently, the contigs were polished based on Illumina short reads using Pilon 1.24 (Walker et al., 2014). The genome completeness was evaluated using BUSCO (version 3.0.2) with fungi_odb10 OrthoDB database (Simao et al., 2015).

The RepeatMasker program (version 4.1.1)^2^ was used for masking repetitive elements in nucleotide sequences of contigs. The genomic structure annotation was performed using BRAKER2 (version 2.1.6) (Bruna et al., 2021), which allows for fully automated training of the gene prediction tools GeneMark-ET (Lomsadze et al., 2014) and AUGUSTUS (Stanke et al., 2006) using RNA-seq data. Microsatellite identification tool (MISA) (Beier et al., 2017) was used to determine the distribution and frequency of various types of simple sequence repeats (SSRs). tRNAs and rRNAs were identified using tRNAscan-SE (version 2.0.7) (Chan and Lowe, 2019) and RNAmmer (version 1.2) (Lagesen et al., 2007), respectively. Other non-coding RNAs, including small RNAs (sRNAs) and small nuclear RNA (snRNAs), were inferred from Rfam using Infernal (version 1.1.4) (Nawrocki and Eddy, 2013).

Functional annotation of genes was conducted by homology searching against protein sequences from the SwissProt,^3^ NR^4^ and eukaryotic orthologous groups (KOG)^5^ databases using Diamond 2.0.9 (Buchfink et al., 2015). For multiple matches of a single protein, only the best match was retained. The gene ontology (GO) annotation was carried out using InterProScan (version 5.45) (Jones et al., 2014). The functional pathway annotation (Kyoto Encyclopedia of Genes and Genomes, KEGG) was carried out using BlastKOALA in KEGG (Kanehisa et al., 2016). Signal peptides and cleavage sites of *C. lacerata* proteins were predicted using SignalP (version 4.1) (Almagro Armenteros et al., 2019). All proteins with signal peptides were analyzed for the presence of transmembrane domains using TMHMM (version 2.0) (Moller et al., 2001). Similarly, Phobius (version 1.01) (Kall et al., 2004),^6^ which predicted the transmembrane structure and signal peptide, was used to expand the subsequent secretory proteins. A protein containing a signal peptide and no transmembrane domain was identified as a secretory protein. The genes encoding Carbohydrate-Active enZYme (CAZyme) (Lombard et al., 2014) were annotated using dbCAN2 (Zhang et al., 2018). The protein sequences of map00900, map00902, map00904, and map00909, which are the major biosynthetic pathways involved in terpenoid synthesis, were downloaded from the KEGG database to evaluate the presence/absence of terpenoid biosynthesis genes.

### Phylogenetic Analysis

The 18 representative genomes of species from the order Polyporales containing both sequences and genomic annotation information were downloaded from NCBI and listed in Table 1. Orthofinder (Emms and Kelly, 2019) and FigTree v1.4.4^7^ were used to compute orthogroups and construct genome-level phylogenetic trees with single-copy orthologous genes based on Maximum Likelihood (ML) algorithm. FastANI (Jain et al., 2018) was used to calculate the whole-genome average nucleotide identity (ANI) with the parameter “fragLen” being set to 1000. Additionally, ITS sequences (Supplementary Material) from 61 representative TYPE materials (Federhen, 2015) of these 18 species were collected from the NCBI fungus ITS project for phylogenetic analysis in MEGA 7 (Kumar et al., 2016), using Neighbor-Joining method with default parameters.

**TABLE 1:** Representative genomes of order Polyporales.

Strain	Assembly accession	Number of scaffolds	Genome coverage	Sequencing technology	Year
*Daedalea quercina* L-15889	GCA_001632345.1	237	144.4 ×	Illumina	2016
*Dichomitus squalens* LYAD-421 SS1	GCF_000275845.1	542	50.63 ×	454; Illumina	2012
*Fibroporia radiculosa* TFFH 294	GCF_000313525.1	861	N/A	N/A	2012
*Fomitopsis pinicola* FP-58527 SS1	GCA_000344655.2	504	85.9 ×	Illumina; PacBio	2013
*Ganoderma sinense* ZZ0214-1	GCA_002760635.1	69	500.0 ×	454; Illumina HiSeq	2017
*Gelatoporia subvermispora* B	GCA_000320605.2	740	56.6 ×	454; Sanger	2013
*Grifola frondosa* 9006-11	GCA_001683735.1	127	100.0 ×	PacBio	2016
*Laetiporus sulphureus* 93-53	GCF_001632365.1	399	85.2 ×	Illumina	2016
*Obba rivulosa*	GCA_001687445.1	712	127 ×	Illumina	2016
*Phanerochaete carnosa* HHB-10118-sp	GCF_000300595.1	1137	58.1 ×	Sanger; 454; Illumina	2012
*Phlebia centrifuga*	GCA_001913855.2	1355	160.0 ×	Illumina HiSeq	2018
*Phlebiopsis gigantea* 11061_1 CR5-6	GCA_000832265.1	573	145 ×	Illumina	2015
*Postia placenta* MAD-698-R-SB12	GCF_002117355.1	549	47 ×	454; Sanger; Illumina	2017
*Trametes cinnabarina* BRFM137	GCA_000765035.1	776	31 ×	N/A	2014
*Trametes coccinea* BRFM310	GCA_002092935.1	222	99.4 ×	Illumina	2017
*Trametes pubescens*	GCA_001895945.1	1731	160.0 ×	Illumina HiSeq	2016
*Trametes versicolor* FP-101664 SS1	GCF_000271585.1	283	40 ×	Sanger; 454; Illumina	2012
*Wolfiporia cocos* MD-104 SS10	GCA_000344635.1	348	40 ×	Sanger; 454; Illumina	2013

## Results

### General Genomic Features of *Ceriporia lacerata* CGMCC No. 10485

The *C. lacerata* genome sequencing yielded 16 Gb of PacBio RS long-read data (∼400 × coverage), 20 Gb of Illumina short-read data (∼500 × coverage), and 12 Gb of Illumina cDNA data for the hybrid assembly and genome annotation. The genome was initially assembled using Canu *via* reads correction, trimming and assembly based on PacBio long-fragment sequences, then polished using Pilon based on Illumina short reads. The final *C. lacerata* genome assembly contained 58 scaffolds with a total consensus genome size of 36,361,585 bp (∼36 Mb) and GC content of 49.33%, which was comparable to the genome sizes of previously sequenced fungi from the order Polyporales (28∼60 Mb). The maximal scaffold size was 4,415,373 bp, and the N50 scaffold size was 3,409.20 kb. The BUSCO evaluation showed 98.4% completeness of this genome, indicating a high-quality genome assembly (Table 2). All annotation statistics are listed in Supplementary Table 1.

**TABLE 2:** Whole-genome assembly features of *C. lacerata* CGMCC No. 10485.

Assembly parameters	Value
Total genome size (bp)	36,361,585
Number of contigs	58
Maximum contig length (bp)	4,415,373
Minimum contig length (bp)	1,192
Average contig length (bp)	626,923
N50 value (bp)	3,409,197
GC (%)	49.33
BUSCO (%)	98.4
**Protein annotation**	
Total number of predicted proteins/genes	13,243
Total number of annotated proteins/genes	9085
Non-coding RNAs	
tRNAs	179
rRNAs	14
snRNAs	10
other	20
**Gene details**	
Number of protein-coding genes	13,243
Average gene length (bp)	1860.07
Gene density (number of genes per Mb)	364.22
**Exon details**	
Number of exons	119,885
Total exon length (Mb)	24.18
Average exon length (bp)	201.71
Average number of exons per gene	9.05
**Intron details**	
Number of introns	89,079
Total intron length (Mb)	6.35
Average intron length (bp)	71.31

A total of 13,243 genes were predicted in the *C. lacerata* genome with an average length of 1,860 bp, 9,085 of which were successfully annotated using the NR, SwissProt, and KOG databases. In addition, 223 non-coding RNAs, including tRNAs, rRNAs, and snRNA were identified in the *C. lacerata* genome. Other information, such as the numbers of exons and introns, is listed in Table 2.

**FIGURE 1:**
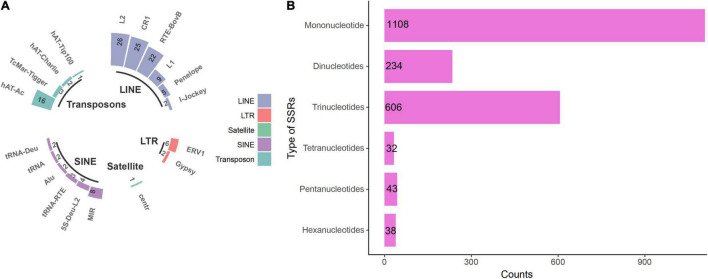
Repetitive sequences in the genome of *Ceriporia lacerata*. **(A)** Repeat region types and counts identified by RepeatMasker. **(B)** SSRs identified by MISA. LINE, long interspersed nuclear element; LTR, long terminal repeat; SINE, short interspersed nuclear element.

Non-coding repeat regions such as microsatellites and simple sequence repeats (SSRs) provide excellent information for assessing genomic variation and are thus frequently used as molecular markers for distinguishing even closely related strains. A total of 5,475 repetitive sequences (277,515 bp) were identified, accounting for 0.76% of the whole genome. More than 81% of these were simple sequence repeats, followed by transposable elements (TEs), including LTRs, LINEs, SINEs, and transposons (Figure 1A). Moreover, 2,061 SSRs were found in the genome (Figure 1B), nearly 54% of which were mononucleotide tandem repeats, followed by tri-, di-, tetra, penta-, and hexanucleotide motifs.

### Functional Annotation

**FIGURE 2:**
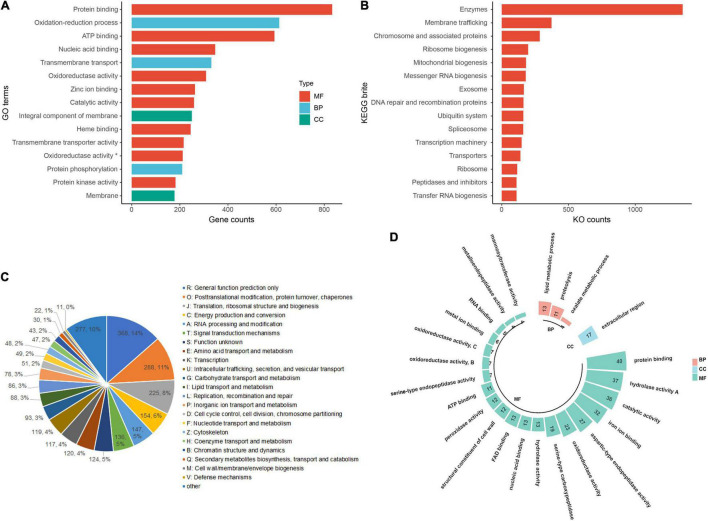
Functional annotations of the *Ceriporia lacerata* genome. **(A)** Top 15 GO terms ranked based on gene counts. GO terms with asterisks refer to oxidoreductase activity acting on paired donors, incorporating or reducing molecular oxygen. **(B)** Top 15 KEGG BRITE categories ranked based on KO counts; **(C)** KOG annotation and classification; **(D)** GO annotations of the secretome. BP, biological process; CC, cellular component; MF, molecular function.

Overall, 6,083 genes (45.9% of the total protein-coding genes) were annotated with GO terms classified as biological process (3,828 genes), molecular function (5,346 genes), and cellular component (1,544 genes), respectively. The top GO terms were protein binding, oxidation-reduction process, ATP binding, nucleic acid binding, zinc ion binding, integral component of the membrane, protein phosphorylation, membrane, regulation of transcription, DNA-templated, carbohydrate metabolic process, nucleus, and host cell nucleus (Figure 2A). In the KEGG annotation of *C. lacerata* genome, 4,215 genes were successfully annotated with 3,170 KEGG Orthologous (KO) terms. These terms were grouped into 42 KEGG BRITE functional hierarchies, including enzymes, membrane trafficking, chromosome and associated proteins, ribosome biogenesis, mitochondrial biogenesis, messenger RNA biogenesis, exosome, DNA repair and recombination proteins, ubiquitin system, and spliceosome (Figure 2B). Both GO and KEGG annotations were mainly concentrated in the basic metabolism and host interactions on which *C. lacerata* depends.

The KOG database is a eukaryote-specific version of the COG (clusters of orthologous groups of proteins) database. Proteins orthologs typically occupy the same functional niche in different species and refer to proteins evolved from vertical families (speciation) from different species. In this study, a total of 2,721 genes (19.0% of the total) were annotated with KOG terms and grouped into 23 classes, generally covering the essential functions of metabolism, genetic information processing, environmental responses, and cellular processes. The top KOG terms were R: General function prediction only, O: Posttranslational modification, protein turnover, chaperones, J: Translation, ribosomal structure and biogenesis, C: Energy production and conversion, as well as A: RNA processing and modification (Figure 2C).

The “secretome” constitutes the entire set of secreted proteins of a microorganism (Ganesan, 2016), including functionally diverse classes of molecules, such as digestive enzymes, chemokines, antibodies, extracellular proteinases, morphogens, toxins, and antimicrobial peptides. For *C. lacerata*, 1,163 genes were found to encode secretory proteins that contain N-terminal signal peptides and no transmembrane domains, accounting for 8.8% of all proteins across the whole genome. Among them, 527 were annotated with GO terms (Figure 2D). Significantly, the top-ranked terms covered a number of secretory digestive enzymes required by this fungus to colonize the woody host and conduct its saprophytic lifestyle, such as protein binding (GO:0005515, protein degradation tagging, cell adhesion), hydrolyzing *O*-glycosyl compounds (GO:0004553, catalysis of the hydrolysis of any *O*-glycosyl bonds for cellulose degradation), aspartic-type endopeptidase activity (GO:0004190), serine-type carboxypeptidase activity (GO:0004185), and proteolysis, serine-type endopeptidase activity (GO:0006508), and metalloendopeptidase activity (GO:0004222). Additionally, a few secretory proteins were involved in a host of diverse and vital biological processes, including calcium ion binding (GO:0005509), zinc ion binding (GO:0008270), signal transduction (GO:0007165), cell surface receptor signaling pathway (GO:0007166), mycotoxin biosynthetic process (GO:0043386), and regulation of cell cycle (GO:0051726), which are related to signal transduction, proliferation, survival, defense, and virulence factors.

### Genome-Level Phylogenetic Analysis of *Ceriporia lacerata* Revealed the High-Resolution Evolutionary Relationships Within the Polyporales

**FIGURE 3:**
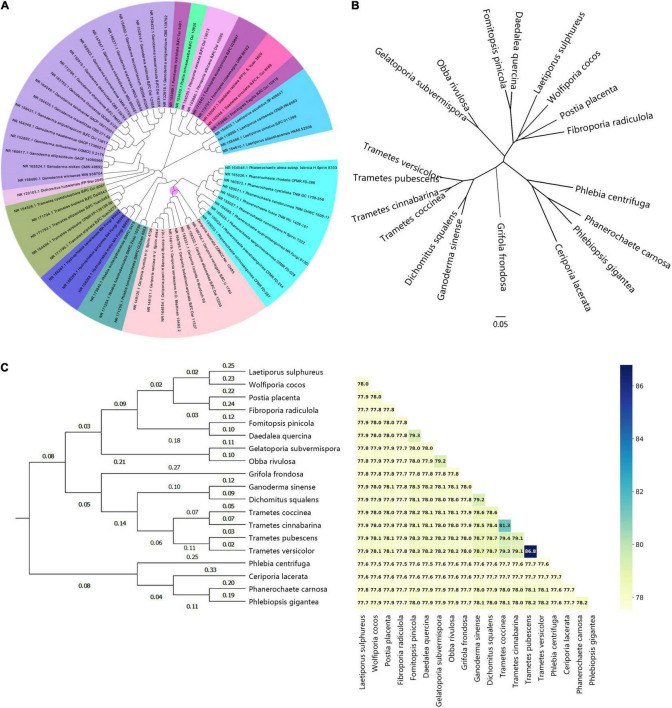
Genome-based phylogenetic analysis of representative strains within the order Polyporales. **(A)** Phylogenetic analysis based on the ITS sequences from representative TYPE materials from Polyporales and *C. lacerata* CGMCC No. 10485. Different genera were distinguished with different colors; **(B)** Unrooted ML phylogenetic tree with single-copy orthologous genes from representative genomes of the order Polyporales based on hidden Markov models. **(C)** ANI values and ANI-value-based hierarchical clustering. The data matrix was created using species as the independent variable and ANI values between two species as the dependent variable using the group-average method and Euclidean distance as a scale.

Even though several studies have been conducted on the phylogeny of *C. lacerata*, most of them were performed using ITS or 18S rDNA sequence alignment (Jia et al., 2013; Miettinen et al., 2016; Ponlada et al., 2016; Wu et al., 2017; Yuan et al., 2017; Chen et al., 2020). In the present study, to reveal the primary phylogenetic position of *C. lacerata* CGMCC No. 10485, a phylogenetic tree was reconstructed (Figure 3A) with 62 ITS sequences (Federhen, 2015) associated with the 19 strains. Collectively, the ITS samples from *Ceriporia, Phlebia*, and *Phanerochaete* were more closely clustered than any other species from the order Polyporales, which was similar to previous reports (Wu et al., 2017; Yuan et al., 2017; Chen et al., 2020). Evidently, *Ceriporia* strains formed a clear monophyletic group, indicating the taxonomic and phylogenetic position of *C. lacerata* CGMCC No. 10485. Subsequently, a denser sampling of larger and orthologous gene sets across the whole genome was conducted to construct more detailed phylogenomic trees to advance the phylogeny and systematics of the order Polyporales.

Genome-based phylogenetic analyses, such as multiple alignment of single-copy orthologous genes (SCOG) and average nucleotide identity (ANI), provide a more robust and reliable taxonomy and phylogeny due to their greater resolution of evolutionary relatedness between different species (Zhang et al., 2017). As shown in Figure 3B, the SCOG phylogenetic tree showed that *Phlebia*, *Ceriporia*, *Phlebiopsis*, and *Phanerochaete* were clustered and merged into an evolutionary branch. ANI is a measure of nucleotide-level genomic similarity, for both complete and draft assemblies, and is a superior indicator of relatedness between the coding regions of two genomes. Variable evolutionary rates as well as the horizontal gene transfer of one or a few genes have no influence on ANI values because the effect of fast-evolving genes is balanced by the slow evolution of other genes (Jain et al., 2018). Generally, an ANI value greater than 95% is usually used as the gold standard for species classification and clustering. According to the results shown in Figure 3C, the ANI values variated between 70 and 80%. This was significantly lower than 95%, confirming that these genomes were from different species. ANI-based clustering indicated that the phylogenetic relatedness between any two genomes was consistent with the SCOG analysis, which further emphasized the robustness and reliability of the phylogenomic analysis shown in Figure 3B.

### Carbohydrate-Active Enzymes of *Ceriporia lacerata* for Lignocellulose and Polycyclic Aromatic Compound Degradation

Carbohydrate-active enzymes (CAZymes) that can act synergistically on a wide range of glycosidic monomers, oligomers, or polymers in a regio- or stereo-specific manner, are required for the breakdown of carbohydrates, along with lignin and hemicellulose. In general, CAZymes include glycoside hydrolases (GHs), glycosyltransferases (GTs), polysaccharide lyases (PLs), carbohydrate esterases (CEs), auxiliary activity proteins (AAs), and carbohydrate-binding modules (CBMs).^8^

**FIGURE 4:**
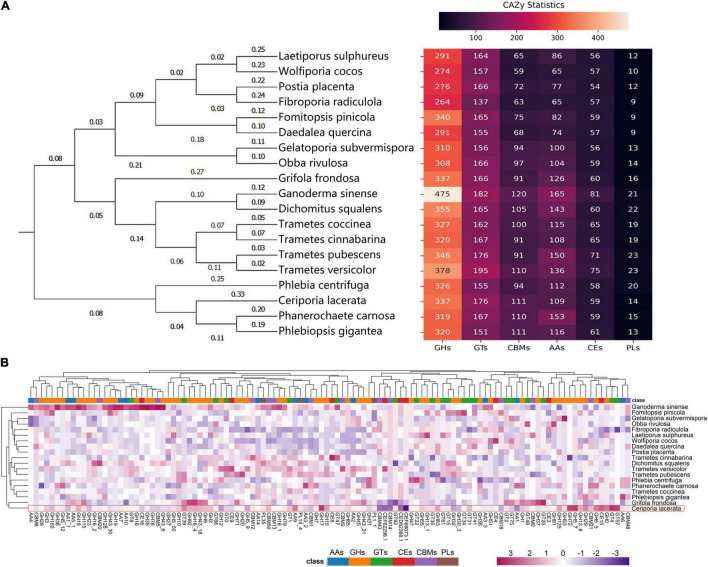
CAZyme-encoding genes in the genome of *Ceriporia lacerata* identified using a comparative approach based on 18 additional species from the order Polyporales. **(A)** Gene counts and phylogenetic analysis of CAZymes. The phylogenetic tree was constructed with ML algorithm. **(B)** Gene count heatmap of CAZyme subfamilies, and the color scale represents the count of the gene normalized by the Z-score method. The CAZyme annotation information of the analyzed species is listed in Supplementary Table 2.

A total of 806 CAZyme-encoding genes were identified in the genome of *C. lacerata*, encompassing 41.8% GHs, 21.8% GTs, 1.74% PLs, 7.32% CEs, 13.5% AAs, and 13.8% CBMs (Figure 4A). Notably, phylogenetic analysis based on CAZyme sequences (the left pane of Figure 4A) was in excellent agreement with the results of SCOG and ANI in Figures 3B,C, indicating the species-specific evolution of CAZymes among Polyporales for their unique saprophytic lifestyle growing on a wood-based matrix.

To evaluate the CAZYme of *C. lacerata* CGMCC No. 10485, comparisons with other 18 fungi of Polyporales were performed. As shown in Figure 4B, CAZymes can be further categorized into subfamilies based on amino acid sequence similarity, substrate selectivity, and catalytic mechanism (Cantarel et al., 2009). Endoglucanases (EC 3.2.1.4), exoglucanases (syn: cellobiohydrolases; EC 3.2.1.91), and β-glucosidases (EC 3.2.1.21) produced by white-rot fungi, are able to break down cellulose synergistically (Daniel, 2016). The endoglucanases in the CAZyme database mainly belong to the GH5∼10 families, while others are classified into GH12, 26, 44, 45, 48, 51, 74, 124, and 148. In the genome of *C. lacerata*, several genes that encode enzymes of GH5, GH7, GH9, GH10, GH12 and GH45 were annotated, among which GH5 (including GH5_5, GH5_50 and GH5_7) and GH9 were more abundant compared with the other species used in this study (Figure 4B). Apart from endoglucanases, several exoglucanases, including GH5, 9, and 51, and β-glucosidases, GH1, 2, 3, 5, 16, and 30 were successfully annotated, demonstrating the potential of this strain to degrade cellulose.

Compared to a wide variety of cellulases with different functions, white rot ligninolytic enzymes are mainly grouped into two families: AA1 [laccase/*p*-diphenol: oxygen oxidoreductase/ferroxidase (EC 1.10.3.2)] and AA2 [manganese peroxidase (EC 1.11.1.13), lignin peroxidase (EC 1.11.1.14), versatile peroxidases (EC 1.11.1.16)] (Mkel et al., 2021). Lignin peroxidases (EC 1.11.1.14) are powerful oxidants with a high redox potential that oxidize lignin’s non-aromatic components. Manganese peroxidase (EC 1.11.1.13) is a Mn-dependent enzyme that can oxidize aromatic substrates but is inactive on non-aromatic lignin. The catalytic activity of manganese and lignin peroxidases are combined in versatile enzymes (EC 1.11.1.16). Laccases (EC 1.10.3.2) are members of the multi-copper oxidase family that catalyze a one-electron oxidation coupled with a four-electron reduction of molecular oxygen to water (Abdel-Hamid et al., 2013). In the genome of *C. lacerata*, 5 and 12 genes were identified as encoding enzymes of AA1 and AA2, respectively. We also discovered a large number of additional AA genes in the genome of *C. lacerata*. They were mainly lytic polysaccharide monooxygenases and enzymes involved in the biodegradation of aromatic compounds, including AA9, AA3_ 2, AA5_ 1, AA10, AA6, AA3_ 3, AA7, AA14, and AA4. Among them, AA4 and AA9 had the greatest gene counts among the selected species (Figure 4B). AA4 proteins are vanillyl-alcohol oxidases that catalyze the conversion of a wide range of aromatic compounds bearing side chains at the para-position of the aromatic ring, while AA9 proteins are copper-dependent lytic polysaccharide monooxygenases responsible for the cleavage of cellulose chains through oxidation of carbons C1, C6, and/or C4. The diversity and abundance of AA families in the *C. lacerata* genome reflects its strong capacity to degrade lignin and various PACs (Mori and Kondo, 2002; Suhara et al., 2003; Xu et al., 2008; Lin et al., 2011; Hong et al., 2012, 2013; Wang et al., 2017; Yanto et al., 2019; Al-Hawash et al., 2020).

### Genetic Basis of Terpenoid Biosynthesis in *Ceriporia lacerata*

**FIGURE 5:**
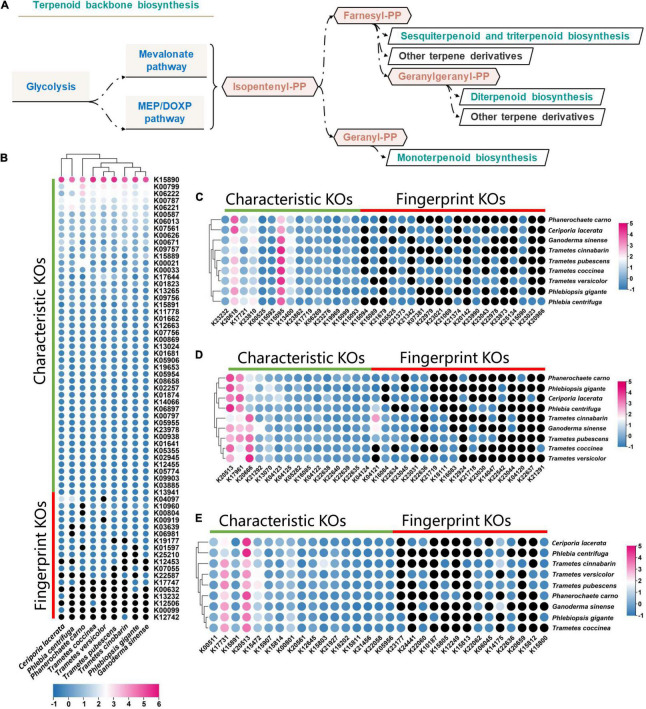
Identification of terpenoid biosynthesis pathways in the *Ceriporia lacerata* genome. **(A)** Sketch of the biosynthesis pathways. **(B)** Annotation of the terpenoid backbone biosynthesis pathway (KEGG pathway map00900); **(C)** Annotation of the monoterpenoid biosynthesis pathway (map00909); **(D)** Annotation of the sesquiterpenoid and triterpenoid biosynthesis pathway (map00904); **(E)** Annotation of the diterpenoid biosynthesis pathway (map00902). The Enzyme Commission (EC) numbers for each pathway were converted into KO identifiers. The gene counts of each KO term are normalized by the Z-score method and increasing from blue to red in the color bar, while the missing KOs are indicated by black circles. More information is supplied in Supplementary Tables 3–6.

Terpenoids, also known as isoprenoids, are categorized by the number and arrangement of carbon atoms generated by the linear arrangement of isoprene units followed by cyclization and rearrangements of the carbon skeleton. The terpenoid backbone is biosynthesized *via* the mevalonic acid (MVA) pathway and the 2-C-methyl-d-erythritol 4-phosphate/1-deoxy-d-xylulose 5-phosphate (MEP/DOXP) pathway (Figure 5A). In the KEGG database, map00900, map00902, map00904 and map00909 are the major biosynthetic pathways that synthesize the backbone of monoterpenoids, diterpenoids, sesquiterpenoids and triterpenoids (Figure 5A). To evaluate the terpenoid synthesis potential of *C. lacerata* CGMCC No. 10485, another 8 genomes, including *Phlebiopsis gigantea, Phlebia centrifuga and Phanerochaete carnosa* (with higher relatedness), as well as the typical terpenoid-producing species *Ganoderma sinense, Trametes versicolor, T. pubescens, T. coccinea*, and *T. cinnabarina* (Ludwiczuk et al., 2017; Winska et al., 2019), were analyzed to annotate their terpenoid biosynthetic pathways. The EC numbers of each gene mapped onto the pathways were converted into KO identifiers.

The annotated KO terms from each pathway are summarized in Figures 5B–E. A total of 131 KO terms involved in terpenoid synthesis were found in the genome of *C. lacerata*. It’s worth noting that all the KOs in each pathway could be classified into two groups based on their presence or absence in the genomes, characteristic KOs that were shared among nine species, and fingerprint KOs that were unique to one or several species.

The MVA and MEP/DOXP pathways make up the main part of map00900. Among the nine genomes, 53∼59 KO terms can be found (Supplementary Table 3). Interestingly, the characteristic KOs were primarily found in the MVA pathway (K00626, K01641, K00021, K00869, K00938, and Figure 5B), farnesyl diphosphate synthesis pathway (K15890, K15891, K05906, K15889, K00587, K06013, and K08658), and geranylgeranyl diphosphate pathway (K05355). However, the absence of downstream strain-specific KOs (K00099, K12506, or K00919) resulted in an incomplete MEP/DOXP pathway, forming the fingerprint area to distinguish the nine species. This finding demonstrated that the MVA pathway was the main determinant of terpenoid synthesis among these white-rot fungi.

According to the KO annotations shown in Figure 5C (map00902, Supplementary Table 4), 22 ∼ 27 KO terms can be found for monoterpenoid biosynthesis pathway. Unique to *C. lacerata* were K21069, K07381, K05525, and K15090, which are predicted to be involved in the biosynthesis of (R)-ipsdienol, (6E)-8-hydroxylinalool, trans-8-oxolinalool, and (-)-trans-carveol, respectively.^9^ In pathway 00904 (Figure 5D and Supplementary Table 5), 20∼ 26 KO terms were annotated. The fingerprint KOs of *C. lacerata* were mainly enriched in the synthetic pathways of ferruginol, 11-hydroxyferruginol and salviol (diterpenoids).^10^ In pathway 00909 (Figure 5E and Supplementary Table 6), 22 ∼ 27 KOs were identified in the nine genomes. Characteristic KOs, K00801 and K00501 are the critical enzymes that successively convert farnesyl diphosphate into triterpenoids. Sesquiterpenes are produced from farnesyl diphosphate *via* one- or two-step catalytic reactions catalyzed by strain-specific enzymes. In this pathway, fingerprint KOs encode various enzymes that depend on the species. In *C. lacerata*, the fingerprint KOs were mainly related to the biosynthesis of nerolidol (sesquiterpene) and germacrene A (triterpenoid).^11^

In conclusion, the characteristic and fingerprint KOs provide an effective identifier to distinguish the main or branching KEGG pathways and are of great importance for understanding strain-specific terpenoid synthesis enzymes.

## Discussion

Most species of Polyporales cause rot in standing trees and fallen logs, which is vital to the nutrient cycling and health of forest ecosystems (Money, 2016). Some Polyporales species, such as *Ganoderma lucidum*, have been employed as natural remedies in traditional Chinese medicine. Purified cell wall polysaccharides and triterpenoids from these fungi offer a wide spectrum of medicinal effects (Boh et al., 2007). Another well-known Polyporales genus is *Trametes*, which is famous for the laccases production in the pulp and paper industry and environmental remediation, as well as the ability to produce bioactive substances (Rodríguez-Couto, 2018). Despite the environmental, pharmaceutical and economic values of such important microbial resources, there are currently insufficient genomic reports on the order Polyporales. At present, only 72 genomes (114 assemblies,^12^ are open access against over a thousand species isolated all over the world, most of them are from the genera *Ganoderma* (Zhu et al., 2015; Tian et al., 2021) and *Trametes* (Pavlov et al., 2015; Lin et al., 2020), while other genera obtained fewer attentions in genomics. Hence, enriching the gene library and screening the candidate functional genes from these species, become more and more urgent.

Given the important potentials of *C. lacerata* in environmental remediation, pretreatment of lignocellulosic biomass for biorefinery, as well as drug R&D, a good reference genome is necessary to characterize the genetic background for mining better lignocellulose degrading enzymes, structurally diverse medicinal secondary metabolites, etc. In this study, by using whole-genome sequencing, hybrid assembly, and comprehensive annotation, a wealth of genetic information about *C. lacerata* was obtained. Based on these data, a comparative genomics analysis was carried out to determine the phylogenic status and the genetic foundations of lignocellulose degradation and terpenoid synthesis with other Polyporales strains.

As for the phylogeny and taxonomy, *Ceriporia* Donk is a genus initially proposed in 1933 (Rajchenberg, 2000), with about 80 species included to date.^13^ The traditional phylogenetic analysis based on morphological, physiological, and biochemical features has provided the backbone of the *Ceriporia* phylogeny. Still, some inherent deficiencies of phenotypic characteristics, such as susceptibility to convergent evolution, reduction, or disappearance, complicate the phylogenetic analysis (Seifried and Temelli, 2009). Hence, a comprehensive phylogenetic analysis of the genus *Ceriporia* integrating morphological characteristics and molecular phylogeny based on the ITS and/or nLSU sequences, was conducted (Jia et al., 2013). More recently, in another study, multilocus single-copy genes provided effective markers to track the evolutionary relatedness of species in the order Polyporales (Binder et al., 2013). The phylogenomic tree based on 356 genes using available Polyporales genome data, generated four well-supported clades, including antrodia (*e.g., Wolfiporia, Fomitopsis)*, gelatoporia (*e.g., Gelatoporia)*, core polyporoids (*e.g., Dichomitus, Ganoderma, Trametes*), and phlebioids (*e.g., Phanerochaete*, *Phlebia*) (Binder et al., 2013). In the present study, the phylogenetic trees based on conserved single-copy genes and ANI from the whole-genome assembly, reproduced the major phylogenetic clades perfectly, clarifying the phylogenetic status of *C. lacerata* within the order Polyporales. The phylogenetic analysis suggested that *C. lacerata* belongs to the phlebioid clade, together with *Phlebia, Phlebiopsis*, and *Phanerochaete*. The phylogeny of Polyporales derived from homology analysis of genetic information greatly facilitates the understanding of their diversity and offers a new perspective on biodiversity conservation.

The capacity to degrade and utilize lignocellulose is one of the most important factors influencing the adaptation of saprophytic fungi to woody habitats. Various fungi obtain their nutrients from primary plant tissues, exudates, or phloem sap, but only a few species can decompose wood effectively (Abdel-Hamid et al., 2013). Fungal cellulases are thought to act together at the sites of wood cell wall degradation. Endoglucanases cleave the backbone of cellulose chains without discrimination, exoglucanases attack cellulose chains from either the reducing or non-reducing ends, while β-glucosidases hydrolyze cellobiose or cello-oligosaccharides to release glucose (Okal et al., 2020). In the present study, most predicted CAZyme-coding genes provided a genetic basis for the saprophytic lifestyle of *C. lacerata* based on lignocellulosic biomass. Several genes encoding endoglucanases, exoglucanases and β-glucosidases were found in the genome of *C. lacerata* and assigned to GH families, supporting the potential of this species to degrade cellulose. During lignin degradation, white-rot fungi generally secrete extracellular lignin-modifying enzymes, the best characterized of which are laccases, lignin peroxidases and manganese peroxidases. The characterized AA1 enzymes (laccases) are multi-copper oxidases that use diphenols and related substances as donors with oxygen as the acceptor, while AA2 family enzymes are class II lignin-modifying peroxidases. It was reported that *C. lacerata* could secrete such enzymes to degrade the softwood of *Pinus densiflora* (Ying et al., 2014a). Moreover, corresponding genes were found in the genome of *C. lacerata*, which confirmed its potential to degrade lignin (Lee et al., 2007). There were up to 109 AA genes, much more than what was found in species from the antrodia clade of Polyporales (*e.g., Wolfiporia, Fomitopsis*), as well as most soft-rot fungi (13∼115), brown-rot fungi (21∼53), and *Trametes versicolor* (89) (Sista Kameshwar and Qin, 2018), demonstrating the strong potential of *C. lacerata* for lignin and PAC degradation.

Terpenoids produced by numerous fungi have attracted increasing attention due to their antimicrobial, anti-inflammatory and antitumor activities. Several species of Polyporales such as *Ganoderma* and *Trametes* (Rajarathnam and Shashirekha, 2003; Ahmad et al., 2021) are naturally excellent terpenoid producers. Similarly, *Ceriporia* species have also been reported to produce a variety of terpenoids, such as α-terpineol, ceriponols A∼K, and lanostane triterpenoids with various bioactivities (Shan et al., 2012; Ying et al., 2013a,b, 2014a,b; Zhao et al., 2013; Lee et al., 2015). The majority of genes encoding enzymes of the terpenoid backbone biosynthesis pathway were found in the *C. lacerata* genome. Characteristic KOs highlight the primary metabolic pathways for terpenoid synthesis, in which the terpenoid backbone synthesis pathway ensures the subsequent production of a wide range of terpenoid structures. Significant variations in terpenoid synthesis of the selected strains were mainly found in map00902, map00904, and map00909, which exhibited the highest strain specificity of their fingerprint KOs. Interestingly, the numbers of KOs or enzymes found in each strain were comparable in spite of the distinct variation in the gene counts of different species. Since *Ganoderma* is being widely investigated as a high-yielding terpenoid producer (Ludwiczuk et al., 2017), which is associated with a much higher abundance of terpenoid-related genes than in any other strains analyzed in this study, it stands to reason that gene counts are associated with terpenoid production ability. Moreover, the fingerprint KOs provide the possibility for the synthesis of new terpenoids. The annotation of terpenoid synthesis pathways provides a theoretical basis to employ Polyporales species, such as *C. lacerata*, to produce active chemicals of various structures in the future.

## Conclusion

To our best knowledge, this is the first report on the *de novo* sequencing, assembly and annotation of the whole genome of *C. lacerata*. The current work revealed the genetic basis for the degradation of lignin, cellulose, and aromatic pollutants by this white-rot fungus, while also addressing its phylogenetic position at the genome-wide level. Furthermore, annotation of genes encoding terpenoid biosynthesis enzymes is critical for understanding the mechanisms behind the production of valuable secondary metabolites, as well as the diversity of main components involved. In-depth research on *Ceriporia* will provide additional resources for the sustainable energy, medical, and agricultural industries in the future.

## Data Availability Statement

The data presented in the study are deposited in the NCBI SRA repository, accession number PRJNA804482 (https://www.ncbi.nlm.nih.gov/bioproject/PRJNA804482).

## Author Contributions

XL and WJ conceived and designed the study. PY and YL performed the experimental work. ZM, HL, YM, DH, and HM performed the data analysis. All authors wrote the manuscript and approved the final manuscript.

## Conflict of Interest

The authors declare that the research was conducted in the absence of any commercial or financial relationships that could be construed as a potential conflict of interest.

## Publisher’s Note

All claims expressed in this article are solely those of the authors and do not necessarily represent those of their affiliated organizations, or those of the publisher, the editors and the reviewers. Any product that may be evaluated in this article, or claim that may be made by its manufacturer, is not guaranteed or endorsed by the publisher.

## Footnotes

1
https://www.ncbi.nlm.nih.gov/bioproject/PRJNA804482

2
http://www.repeatmasker.org/

3
https://www.uniprot.org/

4
https://ftp.ncbi.nlm.nih.gov/blast/db/FASTA/

5
https://ftp.ncbi.nih.gov/pub/COG/KOG/

6
http://phobius.sbc.su.se

7
http://tree.bio.ed.ac.uk/

8
http://www.cazy.org/

9
https://www.genome.jp/kegg-bin/show_pathway?map00902

10
https://www.genome.jp/kegg-bin/show_pathway?map00904

11
https://www.genome.jp/kegg-bin/show_pathway?map00909

12
https://www.ncbi.nlm.nih.gov/genome/?term=Polyporales

13
http://www.mycobank.org/MycoTaxo.aspx
