# Supplementary material for: Whole-Genome Sequencing and Analysis of the White-Rot Fungus Ceriporia lacerata Reveals Its Phylogenetic Status and the Genetic Basis of Lignocellulose Degradation and Terpenoid Synthesis
Source: Front Microbiol. 2022 May 24;13:880946. doi: 10.3389/fmicb.2022.880946 (PMC9171200; doi:10.3389/fmicb.2022.880946)
Supplement: Supplementary file 1 [file Data_Sheet_1.PDF]

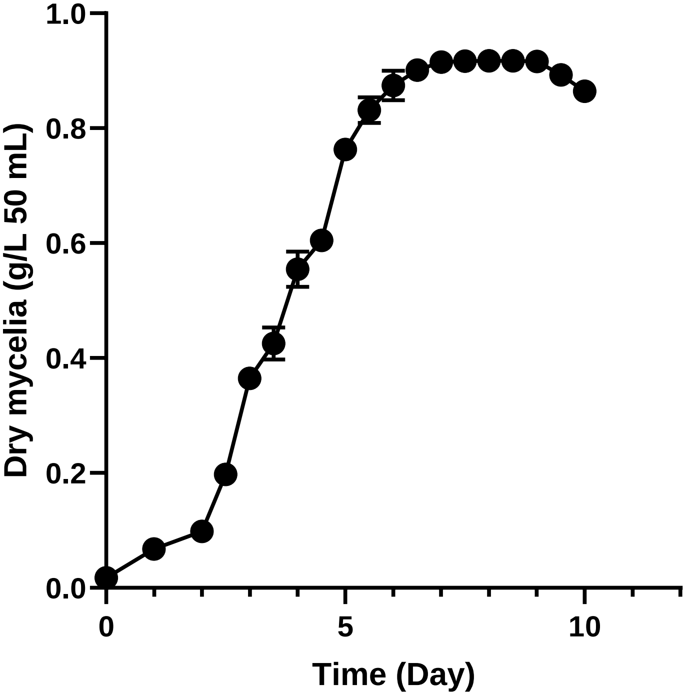

Supplementary Figure S1. Growth curve. Each data is represented by mean±SD caculated from three repetitions.
